# A discussion on the minimum required number of tests in two common pooling test methods for SARS-CoV-2

**DOI:** 10.1017/S0950268821001667

**Published:** 2021-08-03

**Authors:** C. H. Cheng, C. L. Chow, W. K. Chow

**Affiliations:** 1 Department of Architecture and Civil Engineering, City University of Hong Kong, Hong Kong, China; 2 Department of Building Services Engineering, The Hong Kong Polytechnic University, Hong Kong, China

**Keywords:** Pool testing, COVID-19, optimization, pandemic

## Abstract

Pooling of samples in detecting the presence of virus is an effective and efficient strategy in screening carriers in a large population with low infection rate, leading to reduction in cost and time. There are a number of pooling test methods, some being simple and others being complicated. In such pooling tests, the most important parameter to decide is the pool or group size, which can be optimised mathematically. Two pooling methods are relatively simple. The minimum numbers required in these two tests for a population with known infection rate are discussed and compared. Results are useful for identifying asymptomatic carriers in a short time and in implementing health codes systems.

## Introduction

Owing to the recent COVID-19 pandemic, there is a need to test a huge number of people in order to identify the SARS-CoV-2 carriers because many of them are asymptomatic. Even though a number of vaccines have been approved for emergency use, mass screening test is still needed in the near future before COVID-19 is under control. Mass screening test serves to identify asymptomatic carriers and stop potential transmission chains as soon as possible. Such a strategy is especially important in regions where vaccines are short of supply due to various causes, including limited global production capacity, politics and lack of strong global solidarity, all constituting obstacles to equitability in vaccine distribution [1, 2]. In addition to vaccine supply, an in-depth analysis of the unequal distribution of tests, identified cases and fatality rates in 18 most COVID-19-affected countries was recently reported by Shams *et al*. [3]. Furthermore, the effects of the COVID-19 pandemic on daily life were summarised by Haleem *et al*. [4]. Even in regions where vaccine supply is sufficient, the vaccination rate could be low due to poor understanding and hence irrational worrying of the side effects of vaccination among the general public. This is exactly the case in Hong Kong where the COVID-19 full vaccination rate is below 20% as of 12 June 2021 [5]. Mass screening test exerts tremendous pressure on the government in terms of resources and time. One possible way out to more efficiently carry out mass testing [6–14] is to make use of group testing via pooling samples from a group of people, aiming at saving time, materials and test kits. This is especially important in developing countries with limited resources as pointed out before [12–14]. With more efficient use of available resources, a wider testing coverage can be achieved, which is very important in COVID-19 control. By analysing the data in 39 countries, Wei *et al*. [15] showed that testing coverage is an important means of early identification of carriers and hence better control of the disease.

Owing to the high infectivity and high mutation rate of SARS-CoV-2, rapid identification and isolation of individuals infected with the virus via testing would be an ongoing process [8]. However, normal daily life and economic activities were greatly affected [4]. To alleviate such adverse effects, a modified strategy in cutting off the transmission chain of infection is to lock down a building and require the residents in that building for compulsory virus testing when the number of confirmed cases in that building reaches a certain value, as practised in Hong Kong [16]. The threshold number of confirmed cases to elicit lockdown depends on the strain of the virus and on whether the infection source is known. This method is effective, but it usually takes more than 12 h to complete the screening test for buildings in Hong Kong, which are usually tall buildings with large number of residents. Thus it is of paramount importance if the test can be completed in a shorter time so as to restore normal livelihood and business as soon as possible. In this sense, even when resources are not a problem, pooling test is of great value because it saves waiting time. The method used in Hong Kong could be transferred to heavily populated urban areas in big cities.

The method of pooling test has been widely validated for SARS-CoV-2 [17–20] as listed in Pikovski and Bentele [21]. Pooling tests were employed in mass screening of COVID-19 in different countries over the world, including the USA, India, Brazil, China, etc., as summarised in a recent review by Grobe *et al*. [22]. There are many methods for group testing [6–8]. A brief summary on four feasible methods has been recently reviewed [9]. Among these, the simplest method, which is also the pooling method commonly used, was discussed earlier [12–14]. It is interesting to note that pooling test was also studied using mathematical modelling and simulation [23]. Infection rate, test characteristics, population size and testing capacity were used as input parameters and the output fields were the test time and test number required, the number of cases identified and the number of false positives.

In this short communication, another pooling method was studied and compared with the previous one with respect to the minimum number of tests required.

## Two simple pooling test methods

We discussed two of the pooling methods, most attractive by virtue of their simplicity [9] in this paper, with focus on the minimum number of tests required for a given infection rate. These two methods were called Method 1 and Method 2 among the four methods by Mallapaty [9].

In Method 1, the population under test is divided into groups of equal numbers of samples (say *n* in each group). The *n* samples are pooled together and tested. If a group is tested positive, then the *n* samples in that group are tested individually.

Taking an example with one positive case in *m* = 27 samples. By using a group size of *n* = 9, the first round requires three tests and the second round requires nine tests, making a total of 12 *vs.* 27 tests without group testing.

In Method 2, the first round is the same as in Method 1, but the positive group is divided into subgroups of size *k*. Referring to the example of 27 samples, the positive group is divided into three subgroups each containing three samples in the second round. The samples in the positive subgroup are tested individually in the third round of testing. Thus, the total number of tests in the three rounds is 9, further reducing the number of tests.

It should be pointed that in the example above, the number 27 is used to demonstrate the methods in simple numbers. In reality, one positive case in 27 samples is a serious scenario of population infection, meaning that 3.7% of the population is infected. The advantage of pooling test is more pronounced when the infection rate is low, as was shown in the following analysis.

The value of *m*, that is, the average number of samples that contains one positive case, is determined by the current population infection rate θ, with *m* = 1/θ. In Method 1, the total number of tests *L* depends on the value of *n*. In Method 2, *L* depends on *n* and *k.* A problem of practical importance is the choice of *n* (and also *k* in Method 2) to make *L* a minimum. While the solution for Method 1 has been reported by the same authors [13], with *n* = *m*^1/2^ *=* (1/θ)^1/2^, optimisation for Method 2 has not been reported. Thus, it is the aim of this short communication to show how the minimum number of tests required can be achieved via suitable choice of group size in Method 2, and to compare its efficiency with Method 1.

**Fig. 1.**
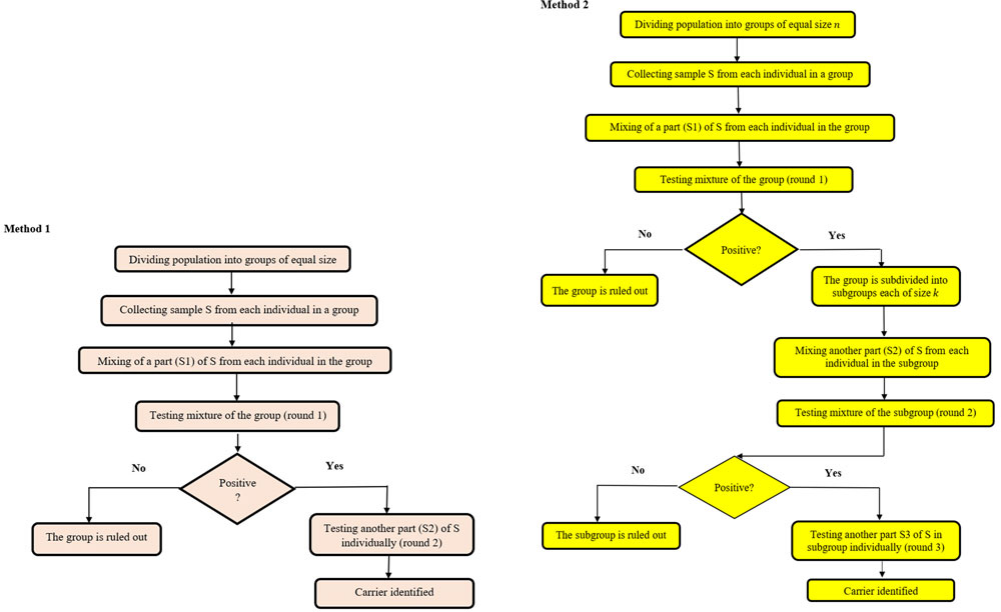
Flowcharts summarizing the procedures in Method 1 and Method 2 in pooling test.

The two pooling test methods discussed above are summarised in the flowcharts in Figure 1.

## Optimisation

Let *P* be the population under test, θ be the current population infection rate. Then most probably there will be one positive case in *m* = 1/θ people. Further, let *n* be the group size in the first round and *k* be the subgroup size in the second round. The total number of tests *L* is given by

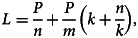


First-order partial derivatives:

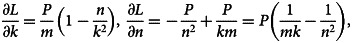


First-order condition for critical points:

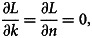

1
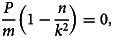

2
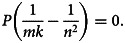


Solving for (1), one has
3
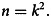


Putting (3) into (2) yields

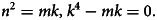


Solving for *k*, one has
4
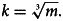


Putting (4) into (3) yields
5
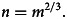


The critical point is 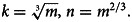


Second-order partial derivatives:
6
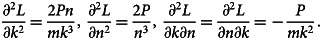


Second-order condition for checking critical points:

Hessian matrix *D* is defined by:
7
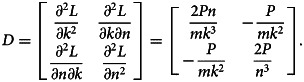


At critical point 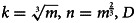
is given by:
8
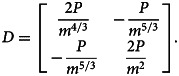


Sign-checking of |*D*|, ∂^2^*L*/∂*k*^2^ and ∂^2^*L*/∂*n*^2^ at critical point 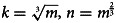:
9
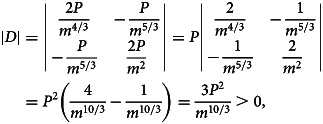

10
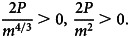


Thus, the critical point 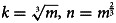 is a local minimum point.

The minimum required number of tests (*L*_min_) in terms of *m* at the critical point 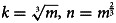 is given by
11
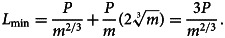


## Comparison of the two methods

A comparison of the two methods is given in Table 1 (for a population of *P*) at a population infection rate of θ and *m* = 1/θ.

**Table 1.** Comparison of the number of tests using different methods

Method	No. of tests *L*	Min. No. of tests *L*_min_
Without grouping	*L*_*o*_ =*P*	*L*_*o*_ =*P*
Method 1 (group size = *n*)	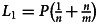	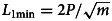 , at 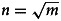 [12]
Method 2 (group size = *n*, subgroup size = *k*)	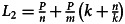	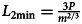 , at 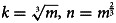

Numerical values are taken with reference to the observed number of infection cases. Taking a higher population infection rate of identifying 1 AC in a building instructed to have mandatory screening of 1000 residents θ = 0.001, *m* = 1/θ = 1000.

Then 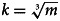 = 10, and *n* = 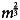 = 100. For a population *P* of 10 million or 10 × 10^6^, the total number of tests using Method 2 is given by Eq. (11):

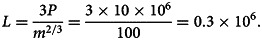


Thus the minimum number of tests required using Method 2 is only 0.03 of the total number of tests without grouping test. For comparison, when using Method 1, the minimum number of tests required is equal to 0.63 × 10^6^, which is 0.063 that without group testing. Method 2 requires less than one-half of the number of tests in Method 1.
12
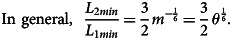


**Fig. 2.**
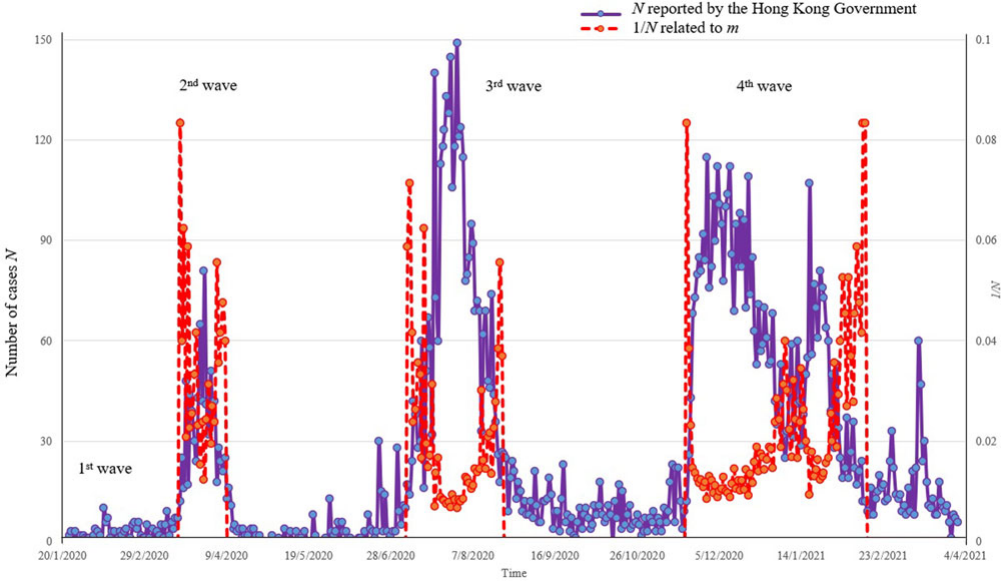
Number of confirmed cases reported by the Hong Kong Government.

As reported by the Hong Kong Special Administrative Region Government [16, 24], there are four waves of infection up to early April 2021 since early 2020. As shown in Figure 2 on the number of daily infection cases *N* in Hong Kong [24], the infection rate θ (related to *N*) could be regarded as 150 in 7 million people (or 0.0002) in the second wave. As *m* is related to 1/θ and θ is *N*/7 × 10^6^, 1/*N* is also plotted in each wave of infection in Figure 2.

**Fig. 3.**
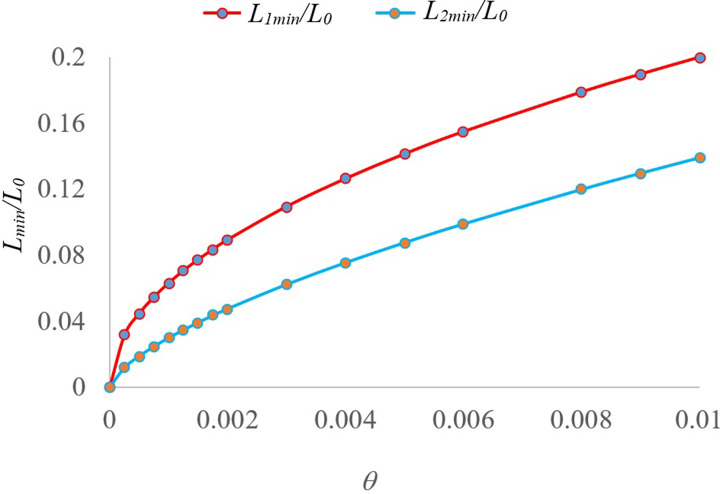
Minimum number of tests required in the two pooling tests relative to that without pooling.

**Fig. 4.**
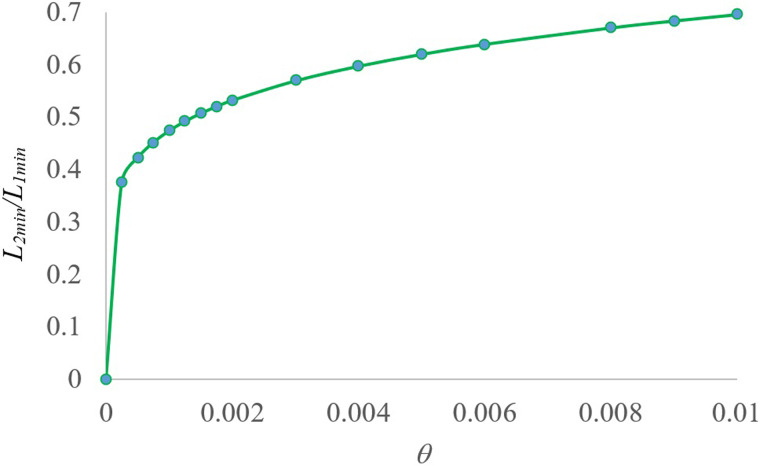
Comparison of the two pooling tests for different infection rates θ.

Equation (12) clearly shows that the advantage of Method 2 over Method 1 in reducing the number of tests becomes more prominent as the population infection rate decreases. That is to say, the saving in resources is larger when the infection rate is low. The dependence of ratio *L*_min_*/L_o_*, where *L_o_* is the number of tests without pooling, on the infection rate θ is shown in Figure 3 for Method 1 and 2. The comparison of *L*_min_ for Methods 1 and 2 is shown in Figure 4. The curves in these figures clearly show the advantage of using Method 2 over Method 1, especially for small population infection rate θ.

Note that in practical cases, 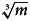 and 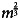 may not be integers, and the integers nearest to 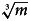 and 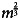 are to be chosen as *k* and *n*.

It should also be pointed out that in the optimisation above, only the mathematical aspect is considered. In practice, other aspects have to be considered. For example, as the group size increases, the reliability of the test results might be compromised due to dilution, and this will increase the likelihood of false-negative test results [8], especially for carriers with low virus load. A balance must be made between increasing the group size and retaining test reliability. In this respect, FDA has provided detailed guidelines for employing pooled sample testing for COVID-19 [25]. The pooling test method is effective and efficient when the positivity rate is low [25].

The robustness of a pooling test method is closely related to the sensitivity and specificity of the test used. In this respect, there is a maximum allowable pool size *n*_max_ below which the pooling test may be considered as robust [26]. Thus to achieve robustness, the optimal pool size to be used should be determined, not only from mathematical optimisation, but in conjunction with the limiting value imposed by *n*_max_. To minimise the influence arising from the uncertainty in prevalence or infection rate, Bish *et al*. [26] also developed a robust pooling strategy via updating pool sizes each week, for each risk group, based on prior week's testing data. This is a more sophisticated approach, though more complex in implementation.

While pooling test is an effective strategy for identifying asymptomatic carriers in mass screening test, symptomatic patients should be dealt with in a different manner in terms of testing for confirmation. In this respect, the point-of-care-testing strategy has been proposed for saving time relative to mass pooling test [14, 27]. Point-of-care (POC) tests are diagnostic tests performed at or near the place where a specimen is collected, such as in hospitals or clinics. Thus transport time of samples to laboratory is minimal, the test is individual, and the symptomatic patient is treated on the spot (POC). Of course, as *a priori* of this strategy, there should be qualified personnel to initially identify the suspected patients before POC testing.

## Conclusions

Pooling tests are commonly used in screening virus carriers. The size of pool is an important parameter as it determines the total number of tests required. A proper choice of group size will yield the minimum number of tests required. Similar to other approaches and methods, there are limitations of the present approach of optimisation and of pooling test methods in general.

### Limitation 1

The optimal group size in pooling test depends on a number of factors, and the present study only considers optimisation with respect to test numbers. Other factors that could affect the optimal group size include the kind of test used and the sensitivity of the test. Thus when considering the optimal group size in pooling test, the results of the present study have to be considered in conjunction with factors such as test sensitivity.

### Limitation 2

As test sensitivity increases, the maximum allowable group size increases. In this paper, we do not consider the upper limit of group size (or equivalently virus concentration in pool) imposed by test sensitivity.

### Limitation 3

Pooling test, be it Method 1 or Method 2, is efficient only when the positivity rate is low because in that case most of the tested cases would be negative and individual testing is not worthwhile and when a large number of people have to be tested in a short time.

### Limitation 4

Pooling test requires two or three rounds of tests, thus requiring a tighter control of logistics to minimise sample handling errors.

### Limitation 5

The results in the present analysis depend on the knowledge of the prevailing infection rate, which is usually changing with time and also not exactly known at the time of test design. An error in *m* will lead to inaccuracy in estimating the minimum number of tests required and hence in testing implementation.

For the two common pooling methods (Method 1, a two-round method, and Method 2, a three-round method), the minimum numbers of tests required are compared in this paper. It is shown that Method 2 is more efficient than Method 1 in reducing the total number of tests, and the reduction increases as the population infection rate decreases. This means that Method 2 requires less cost and resources in terms of test kits and manpower. However, as Method 2 requires three rounds of test, it is more demanding on well-trained personnel in controlling the logistics of the samples. Thus Method 2 should be considered only when a strong team of trained staff members is available.

## Data Availability

Data are available from the authors upon request.
